# Phasor-based analysis of a neuromorphic architecture for microwave sensing

**DOI:** 10.1038/s41598-024-66156-0

**Published:** 2024-07-06

**Authors:** Ashkan Soleimani, Keyvan Forooraghi, Zahra Atlasbaf

**Affiliations:** Department of Electrical and Computer Engineering, Tarbiat Modares University, Tehran, 14115-194 Iran

**Keywords:** Electrical and electronic engineering, Computational science, Design, synthesis and processing

## Abstract

This article presents a design procedure for implementing artificial neural networks (ANNs) using conventional microwave components at the hardware level with potential applications in radar and remote sensing. The main objective is to develop structured hardware design methods for implementing artificial neurons, utilizing microwave devices to create neuromorphic devices compatible with high-frequency electromagnetic waves. The research aims to address the challenge of encoding and modulating information in electromagnetic waves into a format suitable for the neuromorphic device by using frequency-modulated information instead of intensity-modulated information. It also proposes a method for integrating principal component analysis as a dimensionality reduction technique with the implementation of ANNs on a single hardware. As a dummy task, the process outlined here is used to implement an artificial neural network at the hardware level, with a specific emphasis on creating hardware that is capable of performing matrix multiplications in the form of dot products while also being able to extract the resulting data in an interpretable manner. The proposed implementation involves the use of directional couplers to implement weights and sample the resulting signal at specific intervals to obtain the multiplication result.

## Introduction

Most applied artificial neural networks (ANNs) are computer software simulations constructed on electronic von Neumann computer systems. However, the limitations of von Neumann architecture, such as high power consumption, the von Neumann bottleneck, and low computation speed prevent exploiting the maximum potential of ANNs. To address these limitations, more robust alternative architectures naturally matched with ANN topology like neuromorphic and neural network hardware implementations are needed^1–4^.

Neuromorphic computers are a type of non-von Neumann computers that take inspiration from brains to efficiently execute specific tasks they are designed for at accelerated speeds. Their structure and function consist of neurons and synapses which are used for both processing and memory. In contrast, von Neumann computers have separate CPUs and memory units where data and instructions are stored. Programs in neuromorphic computers are defined by the neural network’s structure and parameters, rather than explicit instructions like in von Neumann computers^4^. The use of memristive devices to realize synaptic functionalities offers the opportunity to revolutionize neuromorphic systems with lower cost, lower physical dimensions, and power savings. The majority of implementations currently utilize standard silicon technology that allows for integration with the current metal-oxide-semiconductor (CMOS) technology as well as the wealth of fabrication experience and facilities^5^. Neuromorphic architectures have also been extensively investigated in photonics. The implementations can be categorized based on the optical effect that is exploited to adjust the interconnection weights. Such optical effects include light transmission^6^, diffraction^7^, interference^8^, coupling and scattering^9^.

ANNs are increasingly being used in electromagnetic applications, namely forward and inverse scattering problems^10,11^, numerical methods^12^, antenna design^13,14^, radar and remote sensing^15^, direction of arrival estimation (DoA)^16^, and indoor and outdoor localization^17^. ANNs have been also used to enhance the performance of microwave systems, such as microwave imagers and robotics^18–20^. However, the computation of ANNs is usually carried out on the digital level, which is limited by von Neumann architecture. In many electromagnetic-related classification problems, particularly in radar and remote sensing, the objective is to classify a continuous time-domain signal captured by a sensor or antenna in real-time while consuming the least amount of power. This becomes challenging since most devices rely on batteries as their primary source of power. Therefore, using ANNs can be impractical in some real-world applications. Neuromorphic devices in the microwave region can potentially eliminate this limitation.

In order to feed high-frequency electromagnetic waves as input to a neuromorphic device, it is important to carefully consider the specific characteristics and requirements of the device. The method for feeding such waves may differ depending on the type of device, such as resistive switching devices, ferroelectric transistors, or electric-double-layer transistors in neuromorphic CMOS devices. It is also important to ensure that the chosen input method is compatible with the CMOS manufacturing process, as this enhances reliability, stability, and integration density. Additionally, the input method should be designed to minimize power consumption while maximizing the accuracy of signal processing within the neuromorphic device. Therefore, a separate stage is required to encode and modulate the information in the electromagnetic wave into a format suitable for the neuromorphic device^21–23^.

There have been previous attempts to utilize microwave devices such as metasurfaces^24,25^ or spintronic nano-devices^26^ to develop neuromorphic devices that are compatible with high-frequency electromagnetic waves. For instance, the “learned integrated sensing pipeline” (LISP) aims to provide low-latency and computationally efficient sensors for intelligent systems. LISP combines physical and processing layers, enabling joint optimization of measurement strategies and processing algorithms^24^. Another development in this field is the Programmable Artificial Intelligence Machine (PAIM), which is a programmable diffractive deep neural network that utilizes a multi-layer digital-coding metasurface array to perform various deep learning tasks^25^. While these examples demonstrate the potential of hardware implementations of neural networks in the microwave region, they rely heavily on researchers’ creativity because the requirements for weighting and non-linearity are straightforward, and they can be implemented by a wide range of physical effects. Therefore, there is a need to develop structured hardware design methods for effectively implementing artificial neurons.

This article intends to introduce a systematic design procedure to implement ANNs with conventional microwave components using the relationship between time and frequency domains at the hardware level for electromagnetic signal classification. Furthermore, an additional method is proposed to integrate principal component analysis (PCA) as a dimensionality reduction technique with the implementation of ANNs on a single hardware. To examine and explain the proof of concept, a one-layer neural network with 3 neurons trained on the iris dataset is implemented with microwave components.

**Figure 1:**
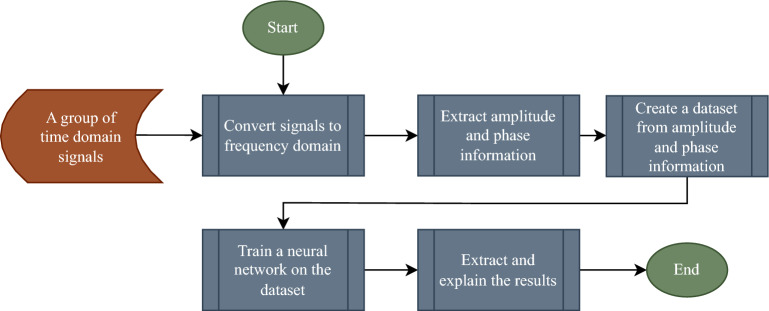
Flowchart of processing time domain signals.

The main difference between this work and other neuromorphic architectures is using frequency-modulated information instead of intensity-modulated information. Figure 1 shows a flowchart of processing time-domain signals, which will be followed here. In this manner, the information is encoded in the amplitude and phase of the signal at each frequency, and the neural network will learn a mapping from data represented in the frequency domain as amplitude and phases of phasors into a space in which the classification explanations will be derived. The main advantage of this process is that it can yield a substantially lower dataset dimension than sampling the time domain data according to the Nyquist rate especially if the signal is composed of a finite and constrained set of countable phasors.

Fundamentally, implementing an artificial neuron at the hardware level is to create hardware capable of performing matrix multiplications in the form of dot product, and extracting the result in an interpretable manner. The proposed method, in this work, is to use directional couplers to implement weights, and sample the resulting signal at periods to obtain the multiplication result.

## Phasor analysis of an artificial neuron

The artificial neuron is the basic processing element in neural networks. It accepts inputs from either the environment or outputs from other neurons. Associated with each input $x_i \in {\mathbb {R}}, i = 1,\dots ,d$ there is a synaptic weight $w_i \in {\mathbb {R}}, i = 1,\dots ,d$, and the output y, is the processed (denoted as function *F*) weighted sum of the inputs $x_i$

1 $$\begin{aligned} y = F\left( \sum _{i=1}^d w_i x_i + w_0\right) \end{aligned}$$

where $w_0$ is the intercept value to make the model more general. It is generally modeled as the weight coming from an extra bias unit $x_0$, which is always +1. In addition, the function *F*, known as the transfer or activation function, is responsible for mapping the input signal to the output signal of the neuron. It is typically chosen from a set of functions that include step, ReLU (Rectified Linear Unit), or sigmoid functions, among others^27^. Performing the summation in the right-hand side of Eq. (1) is the most time-consuming and energy-intensive part of the inference process in neural networks. As a result, the main objective is to introduce hardware that can execute Eq. (1) without consuming power and in real-time.

Time variations in electromagnetic systems often exhibit a cosine wave pattern referred to as time-harmonic. These variations can generally be represented as $e^{j\omega t}$ in which $j=\sqrt{-1}$, $\omega =2\pi f$ represents angular frequency, and *t* is time. The complex forms of the electromagnetic field vectors can be related to their instantaneous field representations using a straightforward method^28^.

In Eq. (2), the signal v(t) is constructed by combining n phasors, $V_i$, where each phasor has an amplitude $A_i$, phase $\theta _i$, and angular frequency $\omega _i$. According to the process depicted in Fig. 1, a neural network will be trained on $A_i$ and $\theta _i$ as inputs. Similar to other classification problems, it is assumed that signals belonging to a class should have similar amplitude and phase distributions.

2 $$\begin{aligned} v(t)=\sum _{i=1}^{n}{\text {Re}}\left[ V_i(x,y,z)e^{j\omega _i t}\right] =\sum _{i=1}^{n}A_i\cos (\omega _it+\theta _i) \end{aligned}$$

Phasors are complex numbers, but although complex-valued artificial neurons exist, real-valued artificial neuron is the main focus of this work. Because of the periodicity of $2\pi$, feeding phases of phasors to a real-valued neuron is challenging. Thus, another approach should be adopted to tackle this inconvenience. Therefore, the initial step is to work with signals having phases of either $0^\circ$ or $180^\circ$ to showcase the implementation process. Subsequently, the influence of arbitrary phases will also be considered. This approach was chosen because it only affects the amplitude of cosine functions.

The signal denoted as *v*(*t*) in Eq. (3) contains phasors with either $0^\circ$ ($A_i>0$), or $180^\circ$ ($A_i<0$) phases and period of *T* which can be calculated by taking the least common multiple (LCM) of the fundamental periodicity of each phasor.

3 $$\begin{aligned} v(t)=\sum _{i=1}^{n}A_i\cos (\omega _it)=A_1\cos (2\pi f_1t)+A_2\cos (2\pi f_2t)+\dots +A_n\cos (2\pi f_nt) \end{aligned}$$

If amplitudes $A_1,A_2,\dots ,A_n$ are fed to a neuron as inputs, the neuron performs a dot product in which the output takes the form of Eq. (1) by replacing $x_i$ with $A_i$. It can be argued that the microwave implemented neuron (MIN) accepts an input in the form of *v*(*t*) in Eq. (3), and returns an output as

4 $$\begin{aligned} v_o(t)= & \, F\left( \sum _{i=1}^{n}w_iA_i\cos (\omega _it)+w_0\right) \nonumber \\= & \, F\left( w_1A_1\cos (2\pi f_1t)+w_2A_2\cos (2\pi f_2t)+\dots +w_nA_n\cos (2\pi f_nt)+w_0\right) \end{aligned}$$

where *F* is the transfer function. Equation (4) at periods or $t=mT$ takes the form of

5 $$\begin{aligned} v_o(mT)= & \, F\left( \sum _{i=1}^{n}w_iA_i+w_0\right) \nonumber \\= & \, F\left( w_1A_1+w_2A_2+\dots +w_nA_n+w_0\right) \end{aligned}$$

Equation (5) describes the expected output of a neuron; thus, if a MIN is fed with a superposition of phasors, the dot product which is used as a criterion for the decision making can be calculated by sampling the output signal at periods.

Generally, a signal can have phases of any value at each frequency of its phasors. Equation (2) takes into account the effects of arbitrary phases, and Eq. (6) formulates the output of a MIN when fed with *v*(*t*) following the same procedure. In this case, the output has additional terms of $\cos (\theta _i)$. Therefore, to account for phase effects, a new dataset can be created using $A_i\cos (\theta _i)$ instead of $A_i$ as inputs, on which the neural network can be trained as formulated in the Eq. (7).

6 $$\begin{aligned} v_o(t)= & \, F\left( A_1w_1\cos (2\pi f_1t+\theta _1)+A_2w_2\cos (2\pi f_2t+\theta _2)+\dots +A_nw_n\cos (2\pi f_nt+\theta _n)\right) \end{aligned}$$

7 $$\begin{aligned} v_o(mT)= & \, F\left( \underbrace{A_1\cos (\theta _1)}_{\text {new input data}}w_1+\underbrace{A_2\cos (\theta _2)}_{\text {new input data}}w_2+\dots +\underbrace{A_n\cos (\theta _n)}_{\text {new input data}}w_n\right) \end{aligned}$$

The mathematical formulation provided the theoretical foundation for understanding the system dynamics. Building upon this formulation, the next step is to translate these equations into practical implementation by considering the circuit parameters. In this manner, weights of the neuron can be viewed as scattering parameters of the microwave hardware. Thus, in one form, the dot product part of a MIN can be realized by a 2-port hardware that relationship between port 1 and port 2 is determined by the trained weights of the neuron. In other words, the dot product part of a 2-port MIN has the scattering matrix in Eq. (8) where $V_1^+$ and $V_2^+$ are incident voltage waves on ports 1 and 2, $V_1^-$ and $V_2^-$ are reflected voltage waves from port 1 and 2, and $s_{11},s_{12},s_{21}$, and $s_{22}$ are scattering parameters.

8 $$\begin{aligned} \begin{bmatrix} V_1^-\\ V_2^-\\ \end{bmatrix}= \begin{bmatrix} s_{11} &{} s_{12}\\ s_{21} &{} s_{22}\\ \end{bmatrix} \begin{bmatrix} V_1^+\\ V_2^+\\ \end{bmatrix} \end{aligned}$$

The $s_{11},s_{12}$, and $s_{22}$ are free parameters, and they can be realized based on the design criteria. For example, both $s_{11}$ and $s_{22}$ can be set to 0 for port matching, and the relation $s_{12}=s_{21}$ can be imposed for reciprocity. But, with the trained weights $w_i$ according to each frequency, the $s_{21}$ parameter should take the following form.

9 $$\begin{aligned} s_{21} = \sum _{i=1}^{n}w_i\delta (f-f_i) \end{aligned}$$

The fact that $w_i$ is a real number dictates that although scattering parameters are complex value numbers in general, the phase of $s_{21}$ should always be $0^\circ$ or $180^\circ$ at $f=f_i$ because the weights are derived from a real-value neuron. A phase correcting stage can be separately designed to ensure this condition. Furthermore, if the input consists of more than one signal, or more than one neuron should be implemented, a n-port MIN can be designed following the same procedure.

The circuit parameters play a crucial role in determining the overall performance of the system. In particular, the relationship between the circuit performance and trained weights becomes evident when examining the system’s behavior under different conditions. In general, the parameter $w_i$ has a wide range of possible values spanning from $-\infty$ to $+\infty$. However, in the context of hardware implementation, $w_i$ can be associated with several meaningful physical interpretations.

- $w_i=0$ indicates that the corresponding phasor has to be eliminated from the calculations. This can be realized by filtering out the signal components corresponding to the frequency of $f_i$, which is analogous to using a band stop filter (BSF).
- $|w_i|=1$ indicates that the corresponding phasor should be transferred with no change in its amplitude, which can be realized by lossless transmission lines or band pass filters (BPFs) in compbination with phase shifters to account for the sign of $w_i$.
- $|w_i| < 1$ indicates that the corresponding phasor is not amplified via the MIN, and only passive components are used. This ensures that energy is not added externally during matrix multiplication. This condition can be achieved by adding a penalty term to the loss function. However, if this leads to low accuracy or underfitting, the neuron can be trained classically and then the weights can be normalized according to the greatest weight. Consequently, the output should be amplified by the same weight or the bias should be normalized accordingly. In the case of using an amplifier, the system is no longer passive.This condition will be referred to as the passive implementation condition (PIC) throughout this work.
- $|w_i| > 1$ indicates that MIN is an active device that consumes energy to perform matrix multiplications. In terms of power consumption, this condition is the least desirable option.

Using this analogy, it is possible to create a device that can perform dot product. This can be done by using conventional microwave devices to create an equivalent microwave circuit or by designing a stand-alone device, such as microwave lenses or antennas, based on the derived scattering parameters. The speed of computation for performing dot product is at the speed of light in the medium of the device, regardless of the method of translating scattering parameters into a microwave neuromorphic device. This is because the designed hardware is an analog device, and the dot product is performed as the electromagnetic wave passes through the device. However, the complexity of the device and the costs and complexity of the manufacturing process can vary based on the type of microwave elements used to implement derived scattering parameters. For example, using metasurfaces instead of regular microwave devices like directional couplers can lead to expensive costs and a much more complex device. The energy consumption is also related to the type of elements used. If passive devices are used, the implemented device would consume no power. But, if active devices like amplifiers are used, the implemented device will consume power to perform multiplications according to the power consumption rate of individual elements. Moreover, if reconfigurable devices like programmable metasurfaces are used, programmability or learnability can also be implemented in the designed neuromorphic device.

When implementing a transfer function, two crucial considerations must be carefully considered. Firstly, the equivalent device should uniformly respond to the entire bandwidth of the output electromagnetic signal, ensuring consistent gain across all frequencies. Secondly, its operation should be influenced by the sampled output voltage. For example, ReLU can be achieved using voltage-controlled switches: when the output voltage is positive, the switch activates, allowing the signal to progress to the next stage; conversely, if the sampled voltage is negative, the switch deactivates, stopping the signal flow. Furthermore, In the case of implementing functions like the sigmoid function, an additional gain corresponding to the transfer function needs to be integrated. Hence, while preserving the core process, alternative implementation strategies may be necessary.

## PCA integration

The complexity of any classifier or regressor affects the number of training samples required to train such a classifier or regressor^29^. This can create problems for neural networks as they may underfit and fail to converge to an acceptable level of accuracy if the number of training samples is small compared to the complexity of the model. In this case, persisting with the learning process may compromise model accuracy on unseen data due to overfitting. Thus, using dimensionality reduction techniques can be helpful, or even crucial in some cases.

One of the most well-known and widely used dimensionality reduction techniques is PCA that aims to find a mapping from the original *d*-dimensional space to a new *k*-dimensional space where $k < d$, while minimizing information loss. The projection of $\textbf{x}$ onto the direction of $\textbf{P}$ is given by $\textbf{x}^\prime =\textbf{P}^T\textbf{x}$, where $\textbf{x}^\prime$ is the projected sample and $\textbf{P}$ is a $d\times k$ matrix found through PCA^29^.

With few exceptions, machine learning algorithms do not perform well when the input numerical attributes have very different scales. Standardization is a common way of making all inputs have the same scale. It first subtracts the mean value from inputs; subsequently, it divides inputs by the standard deviation. The resulting distribution, consequently, always has zero mean and unit variance, and is much less affected by outliers^31^. Standardization is a necessary step without which PCA might yield unreliable results. The process of standardization of a single sample $\textbf{x}$ with mean matrix $\varvec{\mu }=[\mu _1, \mu _2, \dots , \mu _d]^T$ and inverse variance matrix $\left[ \frac{1}{\varvec{\sigma }}\right] =[\frac{1}{\sigma _1},\frac{1}{\sigma _2},\dots ,\frac{1}{\sigma _n}]^T$ can be summarized as

10 $$\begin{aligned} \hat{\textbf{x}} = (\textbf{x}-\varvec{\mu })\odot \left[ \frac{1}{\varvec{\sigma }}\right] = \begin{bmatrix} \frac{x_1-\mu _1}{\sigma _1}\\ \frac{x_2-\mu _2}{\sigma _2}\\ \vdots \\ \frac{x_d-\mu _d}{\sigma _d}\\ \end{bmatrix} \end{aligned}$$

where $\hat{\textbf{x}}$ is the standardized sample, and $\odot$ is element-wise multiplication.

Here, each input represents $A_i$ or $A_i\cos (\theta _i)$ in Eq. (2); thus, another signal called mean signal, denoted by *m(t)*, can be created whose phasor’s amplitudes are $\mu _1, \mu _2, \dots ,\mu _d$. The mean signal can be calculated as

11 $$\begin{aligned} m(t)=\sum _{i=1}^{n}\mu _i\cos (2\pi f_it)=\mu _1\cos (2\pi f_1t)+\mu _2\cos (2\pi f_2t)+\dots +\mu _n\cos (2\pi f_nt) \end{aligned}$$

After performing PCA, and training the neural network on the reduced data, the output of the neuron can be formulated for each sample as

12 $$\begin{aligned} y = \left( (\textbf{x}-\varvec{\mu })\odot \left[ \frac{1}{\varvec{\sigma }}\right] \right) \times \textbf{P}^T\times \textbf{w}+w_0 =(\textbf{x}-\varvec{\mu })\times \underbrace{\left( \left[ \frac{1}{\varvec{\sigma }}\right] \odot (\textbf{P}^T\times \textbf{w})\right) }_{\mathbf {w^\prime }}+w_0 \end{aligned}$$

where

13 $$\begin{aligned} \mathbf {w^\prime } = \left[ \frac{1}{\varvec{\sigma }}\right] \odot [\textbf{P}^T\times \textbf{w}] =\begin{bmatrix} \frac{1}{\sigma _1}\\ \frac{1}{\sigma _2}\\ \vdots \\ \frac{1}{\sigma _d}\\ \end{bmatrix}\odot \begin{pmatrix} \begin{bmatrix} p_{11} &{} p_{12} &{} \dots &{} p_{1d}\\ p_{21} &{} p_{22} &{} \dots &{} p_{2d}\\ \vdots &{} \vdots &{} \ddots &{} \vdots \\ p_{k1} &{} p_{k2} &{} \dots &{} p_{kd}\\ \end{bmatrix}^T\times \begin{bmatrix} w_{1}\\ w_{2}\\ \vdots \\ w_{k}\\ \end{bmatrix} \end{pmatrix} = \begin{bmatrix} \frac{\sum _{i=1}^{k}p_{i1}w_i}{\sigma _1}\\ \frac{\sum _{i=1}^{k}p_{i2}w_i}{\sigma _2}\\ \vdots \\ \frac{\sum _{i=1}^{k}p_{id}w_i}{\sigma _d}\\ \end{bmatrix} \end{aligned}$$

The $\mathbf {w^\prime }$ matrix in Eq. (13) should be calculated before hardware implementation. It is of utmost importance to note that in this case, the PIC should not be forced on neural network’s learning process which yields $\textbf{w}$, but rather, it should be imposed on $\mathbf {w^\prime }$; consequently, if it does not follow the PIC, meaning that the $\mathbf {w^\prime }$ matrix has at least one element that its absolute value is greater than 1, the $\mathbf {w^\prime }$ matrix can be normalized according to its greatest element. If the greatest element in $\mathbf {w^\prime }$ or $max(|w_j|)$ is denoted by $w_m$, the normalized $\mathbf {w^\prime }$ or $\widetilde{\textbf{w}}$ matrix can be written as

14 $$\begin{aligned} \widetilde{\textbf{w}} = \frac{1}{w_m}{\textbf{w}}^\prime = \left[ \widetilde{w}_1,\widetilde{w}_2,\dots ,\widetilde{w}_d\right] ^T = \left[ \frac{\sum _{i=1}^{k}p_{i1}w_i}{\sigma _1w_m}, \frac{\sum _{i=1}^{k}p_{i2}w_i}{\sigma _2w_m}, \dots , \frac{\sum _{i=1}^{k}p_{id}w_i}{\sigma _dw_m}\right] ^T \end{aligned}$$

After normalization, the effect of $w_m$ should be taken into account. This can be achieved by either comparing the output of the designed device with normalized bias or $w_0/w_m$, or amplifying the output by $w_m$, or a combination of both.

**Figure 2:**
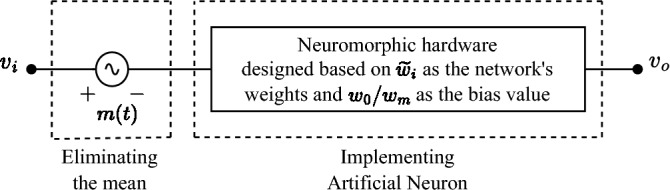
PCA integrated neuromorphic hardware.

The method of implementing a neural network from scattering parameters can be modified to integrate PCA. Figure 2 shows the implemented neuron with PCA integration, in which the following modifications are performed on the primary design

1. A source that creates signal *m*(*t*) was added as a part of the standardization process in PCA. It should be synchronized with the received signal $v_i$.
2. The design of neuromorphic device is based on normalized reduced wights ($\widetilde{w}_i$).
3. As discussed previously, an amplifier may or may not be placed after the power combiner to compensate the effect of normalizing weights according to the greatest one, for which the decision is entirely based on the corresponding problem. It is worth noting that due to the creation of signal *m*(*t*), the overall circuit is not passive.

**Figure 3:**
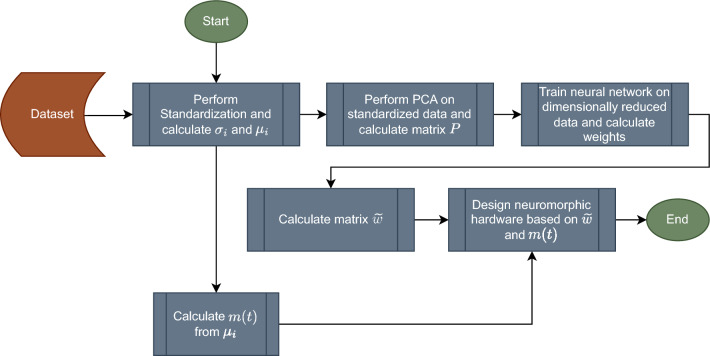
Flowchart of integrating of PCA into neuromorphic hardware.

The process of integrating PCA can be summarized in the flowchart of Fig. 3.

## Experiment

To showcase the proposed design procedure, the Fisher’s 1936 iris flower classification is chosen as a dummy task, given its reputation as a well-known machine learning classification benchmark. This dataset contains 150 different flower specimens, each with 4 variables representing the dimensions of specific features, namely sepal length (SL), sepal width (SW), petal lengths (PL), and petal widths (PW) of multiple iris flower specimens. The dataset also includes three iris species classes, namely Setosa, Virginica, and Versicolor^32^. With these variables as inputs, we can create an architecture that can classify the 150 iris flowers into their respective 3 species.

To start, the values of the iris dataset should be modulated into a waveform by altering the amplitude of the four variables using four phasors with different frequencies, as shown in the Eq. (15).

15 $$\begin{aligned} v(t)=A_{SL}\cos (2\pi f_1t)+A_{SW}\cos (2\pi f_2t)+A_{PL}\cos (2\pi f_3t)+A_{PW}\cos (2\pi f_4t) \end{aligned}$$

**Figure 4:**
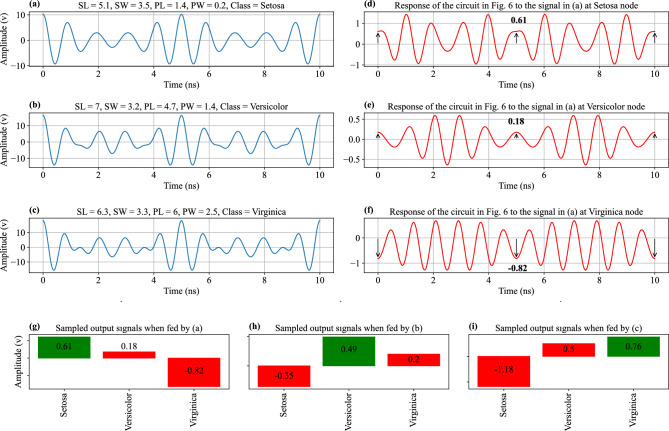
Iris Sample Signals and Circuit Response. This figure presents the iris sample signals for (**a**) Setosa, (**b**) Versicolor, and (**c**) Virginica. It also demonstrates the response of the circuit in Fig. 7 to the Setosa sample signal in (**a**) at the output nodes for (**d**) Setosa, (**e**) Versicolor, and (**f**) Virginica. Additionally, the figure illustrates the sampled voltage of the output nodes when they are stimulated with sample signals from (**g**) Setosa, (**h**) Versicolor, and (**i**) Virginica, which correspond to the signals shown in (**a**), (**b**), and (**c**), respectively.

The variables $A_{SL}$, $A_{SW}$, $A_{PL}$, and $A_{PW}$ represent the corresponding inputs, while $f_1$, $f_2$, $f_3$, and $f_4$ denote the phasor frequencies, which are 1 GHz, 1.2 GHz, 1.4 GHz, and 1.6 GHz, respectively. These frequencies collectively contribute to the signal periodicity with a period of 5 ns. It is important to note that these frequencies were selected based on our discretion, and they are not predetermined or forced. This is because although the data have to be modulated in a signal for this particular case, in electromagnetic classification problems, the data are automatically modulated in such signals. Samples of the modulated voltage waves from each class can be seen in Fig. 4a,b,c.

It is essential to note that every neuron in the network must have the same specific input value. Without this, the neural network’s results will not be trustworthy. While it is easy to achieve this in software implementations, it requires careful attention in hardware implementations. Unwanted reflections can cause imbalances in the input signals received by each MIN. To ensure that the input is equally distributed and separated across all MINs, a power divider can be used. This component divides and allocates the input data into equal segments. To account for its scattering behavior, a new dataset should be derived from the original iris dataset. In the new dataset, each input value is divided by the $\sqrt{3}$.

Using the backpropagation method, a layer consisting of 3 neurons was trained with the sigmoid as transfer function. Afterward, all weights are normalized according to the greatest weight which is $-2.111022$ to include PIC by dividing all weights by its absolute value (2.111022). Table 1 displays the calculated and implemented weights, bias, and accuracy in each case. For implementation, weights with values such as $|w_i|\simeq 1$ or $w_i\simeq 0$ are rounded for simplicity of implementation. The accuracy values in Table 1 indicate that variations in weight values do not affect classification accuracy as long as the relationship between the output node values remains unchanged and the node with the highest value determines the class. Therefore, we can normalize the weights to the highest value node, which is useful when using special microwave devices like passive devices. This also suggests that small variations in implemented weights during manufacturing have negligible effects on the overall accuracy of the system.

It is important to note that the sigmoid function exhibits a strictly increasing behavior. Consequently, despite its utilization in the training phase, and given that the network in question is a shallow one-layer network, it can be disregarded during the implementation stage. Instead, the focus can be exclusively directed towards the values of the implemented nodes for the purpose of classification. In other words, the node possessing the highest positive value is attributed as the representative class without implementation of sigmoid function at hardware level.

**Table 1:** Trained, normalized and implemented weights of the neural network trained on the iris dataset.

	Neuron	$w_1$	$w_2$	$w_3$	$w_4$	bias ($w_0$)	Accuracy
		$f_1=1$ GHz	$f_2=1.2$ GHz	$f_3=1.4$ GHz	$f_4=1.6$ GHz		
Trained	1	0.9915903	0.22570407	−1.61422	−2.111022	0.9338571	0.967
	2	0.34843984	−0.36651236	0.3228167	0.15259588	−0.26222828	
	3	0.03108817	−1.4163296	1.1181797	1.9439662	−1.1336281	
Normalized	1	0.469720496	0.106916967	−0.764662803	−1	0.25540361	0.967
	2	0.165057418	−0.173618446	0.152919629	0.07228531	−0.07171766	
	3	0.014726597	−0.670921288	0.529686427	0.920864965	−0.31003963	
Implemented	1	0.5	0	−0.8	−1	0.6	0.967
	2	0.2	−0.2	0.15	0	0	
	3	0	−0.7	0.5	1	−0.9	

**Figure 5:**
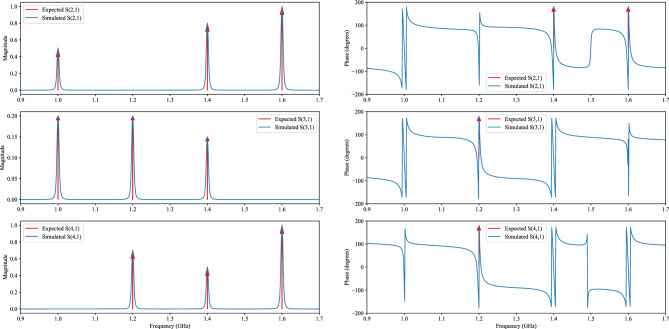
Expected and simulated scattering parameters of a neuromorphic device for classifying iris dataset.

The derived calculations suggest that a neuromorphic device tailored for iris dataset classification requires four ports, with one designated for input and the other three allocated for each class within the dataset. These port configurations correspond to the specific scattering parameter values outlined in Table 1. According to Eq. (9) the expected relevant scattering parameters of the device with ideal power combiners are represented by Eq. (16) through (18), and they are visually depicted with separate magnitude and phase plots in Fig. 5, where negative values are represented with $180^\circ$ phase.

16 $$\begin{aligned} s_{21}= & \, 0.5\delta (f-1)-0.8\delta (f-1.4)-\delta (f-1.6) \end{aligned}$$

17 $$\begin{aligned} s_{31}= & \, 0.2\delta (f-1)-0.2\delta (f-1.2)+0.15\delta (f-1.4) \end{aligned}$$

18 $$\begin{aligned} s_{41}= & \, -0.7\delta (f-1.2)+0.5\delta (f-1.4)+\delta (f-1.6) \end{aligned}$$

A microwave hardware can be designed in various ways to meet the expected scattering parameters in Eqs. (16) through (18). However, a simpler way to design it is to use three separate 2-port MINs instead of a single 4-port microwave device. To implement a 2-port MIN, directional couplers can be used since they affect the amplitude of phasors at each frequency, thereby eliminating the need for the phase correcting stage. A directional coupler is a 4-port microwave component in which the power applied to port 1, is transferred to port 3 (the coupled port) with a coupling factor of $C^2$ ($0<C<1$), while the remaining input power is directed to port 2 (the through port) with a coefficient of $D^2=1-C^2$. In an ideal directional coupler, no power is transmitted to port 4 (the isolated port)^30^.

**Figure 6:**
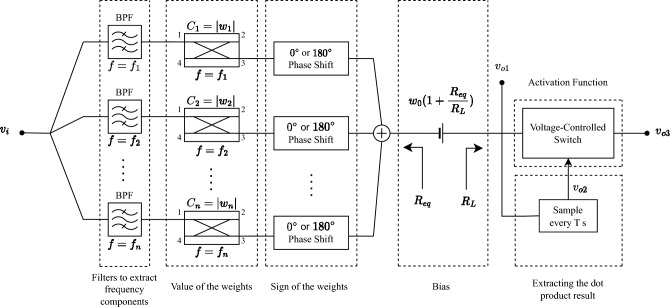
Hybrid coupler network as a neuron.

The idea here is to use the coupling effect as a weight in a neuron, or $w_i = C_i$ where $w_i$ is a weight and $C_i$ is the corresponding coupler’s coupling factor. For each weight, a specifically designed coupler can be utilized. If $v_i(t)$ is assumed as the received signal of the hybrid coupler network of Fig. 6, it can act as a MIN in which every coupler corresponds to a weight that is the amplitude of the corresponding phasor at the corresponding frequency, and phase shifters or $\lambda /2$ or $\lambda$ transmission lines can be used to apply the sign of weights. A voltage-controlled switch is used after the battery to replicate the ReLU transfer function of the neuron. Howerver, it can be omitted for the iris network classifier as discussed before.

Since $0<C_i<1$, it is crucial to train the neural network with respect to PIC. Furthermore, when $|w_i|\simeq 1$ or $w_i\simeq 0$, a coupler is not necessary, as band pass and band stop filters can be used instead. A DC battery can be utilized to incorporate the bias of the neuron. The battery’s value is appropriately scaled by the factor of $1+R_{eq}/R_L$ to account for its distribution between the load resistance ($R_L$) and the equivalent resistance observed at the output of the power combiner ($R_{eq}$).

If *v*(*t*) in Eq. (3) is fed to the hybrid coupler network of Fig. 6, the output $v_{o1}$ is

19 $$\begin{aligned} v_{o1}=w_1A_1\cos (2\pi f_1t)+w_2A_2\cos (2\pi f_2t)+\dots +w_nA_n\cos (2\pi f_nt)+w_0 \end{aligned}$$

which at periods or $t=T$ is equivalent to $v_{o2}=A_1w_1+A_2w_2+\dots +A_nw_n+w_0$ that has the same form of Eq. (1). Therefore, by sampling $v_{o1}$ at periods, a single neuron can be implemented using a network of directional couplers, microwave filters and phase shifters. This architecture, known as hybrid coupler network, is incredibly easy to design and fabricate, and the costs associated with manufacturing it are significantly low.

**Figure 7:**
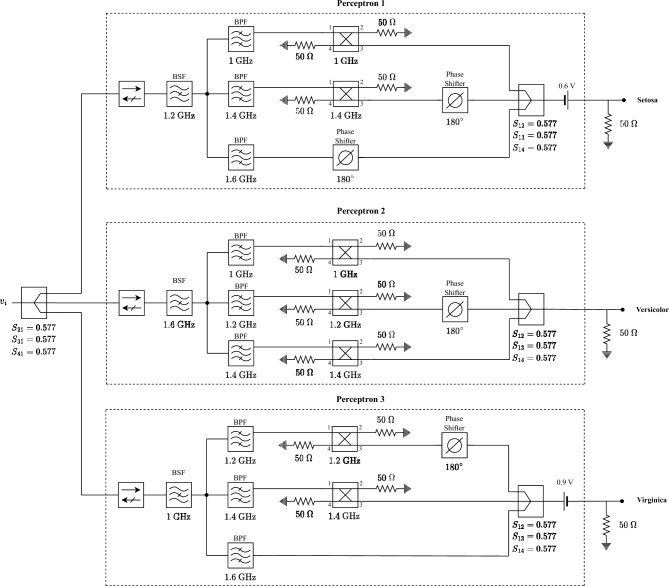
Microwave implementation of neural network with 3 neurons at the output layer.

Figure 7 illustrates the suggested microwave design for the neural network based on hybrid coupler network. It uses suitable impedances for port matching, isolators to prevent undesirable reflections and separate MINs, band pass and band stop filters to gather data points at specific frequencies, and phase shifters to incorporate the negative weight values. Considering the utilization of power combiners at the termination of each branch to combine the individual voltage waves, it becomes imperative to account for their associated gain. This can be achieved by appropriately dividing the bias based on the scattering parameters of the power combiner. Referring to Fig. 7, it is essential to divide each bias by a factor of $\sqrt{3}$. Thus, the biases in the normalized section of Table 1 are also divided by $\sqrt{3}$. In addition, the biases also scaled to include DC voltage distribution between $R_{eq}$ and $R_L$ in implemented section of Table 1. The scattering parameters of the circuit in Fig. 7 are shown in Fig. 5 with blue lines in which the power combiners are considered ideal. In this case, nonideal power dividers or power combiners would result in a consistent decrease across all nodes and frequencies, ensuring a consistent relationship between the output values at each node.

When *v*(*t*) in Eq. (15) is given to the circuit shown in Fig. 7, the output signals at the Setosa, Versicolor and Virginica nodes take on the form of $v_{o1}$ in Eq. (19). This output matches the output of the trained neurons when $t=nT$. By sampling the output signals every 5 ns, the neural network’s output can be obtained, and ultimately solve the classification problem. The node with the highest sampled voltage determines the class.

Figure 4d,e,f show output signals at the Setosa, Versicolor, and Virginica nodes when the circuit is fed with the sample signal in Fig. 4a, which belongs to the Setosa class. At a time of $t=5$ ns, the voltage at the Setosa, Versicolor, and Virginica nodes are 0.61, 0.18, and -0.82 volts, respectively. Based on this, the input signal is classified as Setosa, which has the highest voltage. Figure 4g,h,i show the output signals at the Setosa, Versicolor, and Virginica nodes when fed with signals in Fig. 4a through c, respectively, with the classified signal having the highest voltage value.

When comparing this work to similar works in^33,34^, it becomes clear that there are distinct differences in approaches and performances. The hybrid coupler network relies on passive components such as directional couplers, microwave filters, and phase shifters for computation. This leads to no power consumption for matrix multiplications since all the required power is derived from the input signal’s power. Passive devices typically demonstrate this type of performance. Nonetheless, when compared to similar devices like memristors, passive devices are significantly easier to design and manufacture. Moreover, the costs related to producing passive devices are much lower than those associated with memristors. While it is not reconfigurable or programmable, it can perform calculations of a shallow neural network independent from hardware. In other words, even though the footprint scales with input dimension, energy consumption, and calculation speed are not dependent on it. PCA also can be integrated into the hybrid coupler network; however, although the speed of calculation is not affected by the integration, the power consumption will scale according to the mean value of inputs. In addition, the data are transmitted through electromagnetic signals, eliminating the need for data modulation for electromagnetic classification problems resulting in an end-to-end system; however, for other problems, data modulation is necessary. The hybrid coupler network is cost-effective and easy to fabricate. It achieved an accuracy rate of 96.7% on the iris dataset.

In contrast^33^, uses ONNs (Optical Neural Networks) in the photonic domain, and uses light intensity modulated data. It is a one-layer network, with calculation speed limited by electrical equipment but it can be accelerated with high-speed programmable modulators and detectors. It uses ultracompact diffractive cells and slab waveguides to replace Mach–Zehnder interferometers (MZIs). The energy consumption and footprint scale linearly, and it operates with low power in the mW range. It also has an accuracy rate of 96.7% on the iris dataset.

^34^ utilizes vertical-cavity surface-emitting lasers (VCSELs) and photonic spiking neural networks (SNNs) with a larger number of nodes. It is highly hardware-friendly, inexpensive, and operates at low power (sub-mW). It provides full control over the number of interconnected spiking nodes and has a higher accuracy rate of over 97% on the iris dataset. Data modulation occurs through the intensity of light.

These comparisons highlight the differences in technological approaches and performance characteristics including factors such as reconfigurability, programmability, network depth, speed limitations, energy consumption, footprint scalability, cost, accuracy on the iris dataset, and data modulation methods.

## Discussion

Real-time and low-power inference is crucial in some applications such as real-time control, real-time digital image reconstruction, and autonomous robot control, which with the state of the art von Neumann’s computer architecture is difficult to achieve since von Neumann architecture is naturally very power-hungry and slow. This makes the use of neural networks very limited. In this article, a systematic design procedure for implementing a neural network using conventional microwave components is introduced for electromagnetic signal classification. The approach involves utilizing the relationship between time and frequency domains at the hardware level to derive scattering parameters of the equivalent device. These parameters can then be implemented using a wide range of microwave elements, each with its advantages and disadvantages. Additionally, a method to integrate PCA as a dimensionality reduction technique with the implementation of neural network on a single hardware is proposed. To demonstrate the proof of concept, a one-layer neural network with 3 neurons was trained on the iris dataset, ans an equivalent circuit is designed using microwave components such as directional couplers, phase shifters, power dividers, and power combiners. The hybrid coupler network is capable of solving binary and multi-class classification problems by performing dot product. While it was originally designed for a single neuron, it can also be used to implement neural networks. The focus of this method is on implementing the dot product, which has been successful in delivering a system with the significant advantages of low power consumption and ultra-fast speed. This enables real-time inference with greater power efficiency. Additionally, integrating PCA enhances the method’s ability to effectively solve complex problems with improved accuracy and efficiency.

## Methods

In this work, python programming language and Keras API are used for all the neural network’s training processes, and electromagnetic numerical simulations are conducted in ADS software using harmonic balance simulation.

## Data Availability

Data will be made available on request from the corresponding author.
